# Targeting the *Pseudomonas aeruginosa* Virulence Factor Phospholipase C With Engineered Liposomes

**DOI:** 10.3389/fmicb.2022.867449

**Published:** 2022-03-18

**Authors:** Heidi Wolfmeier, Samuel J. T. Wardell, Leo T. Liu, Reza Falsafi, Annette Draeger, Eduard B. Babiychuk, Daniel Pletzer, Robert E. W. Hancock

**Affiliations:** ^1^Department of Microbiology and Immunology, Centre for Microbial Diseases and Immunity Research, University of British Columbia, Vancouver, BC, Canada; ^2^Institute of Anatomy and Cell Biology, Paracelsus Medical University, Salzburg, Austria; ^3^Department of Microbiology and Immunology, University of Otago, Dunedin, New Zealand; ^4^Institute of Anatomy, University of Bern, Bern, Switzerland

**Keywords:** cholesterol, sphingomyelin, *plcH*, abscess, dermonecrosis, anti-virulence

## Abstract

Engineered liposomes composed of the naturally occurring lipids sphingomyelin (Sm) and cholesterol (Ch) have been demonstrated to efficiently neutralize toxins secreted by Gram-positive bacteria such as *Streptococcus pneumoniae* and *Staphylococcus aureus*. Here, we hypothesized that liposomes are capable of neutralizing cytolytic virulence factors secreted by the Gram-negative pathogen *Pseudomonas aeruginosa*. We used the highly virulent cystic fibrosis *P. aeruginosa* Liverpool Epidemic Strain LESB58 and showed that sphingomyelin (Sm) and a combination of sphingomyelin with cholesterol (Ch:Sm; 66 mol/% Ch and 34 mol/% Sm) liposomes reduced lysis of human bronchial and red blood cells upon challenge with the *Pseudomonas* secretome. Mass spectrometry of liposome-sequestered *Pseudomonas* proteins identified the virulence-promoting hemolytic phospholipase C (PlcH) as having been neutralized. *Pseudomonas aeruginosa* supernatants incubated with liposomes demonstrated reduced PlcH activity as assessed by the *p*-nitrophenylphosphorylcholine (NPPC) assay. Testing the *in vivo* efficacy of the liposomes in a murine cutaneous abscess model revealed that Sm and Ch:Sm, as single dose treatments, attenuated abscesses by >30%, demonstrating a similar effect to that of a mutant lacking *plcH* in this infection model. Thus, sphingomyelin-containing liposome therapy offers an interesting approach to treat and reduce virulence of complex infections caused by *P. aeruginosa* and potentially other Gram-negative pathogens expressing *PlcH*.

## Introduction

The Gram-negative bacterium *Pseudomonas aeruginosa* is one of the most persistent nosocomial pathogens and frequently causes substantial morbidity and mortality, especially in immunocompromised patients (Strateva and Yordanov, 2009; Nguyen et al., 2018). It is associated with acute and chronic lung infections, colonization of burns, wounds, and medical devices, blood borne infections, the formation of abscesses, and many other medical problems (Lister et al., 2009; Gellatly and Hancock, 2013). Its recalcitrant behavior and multidrug resistance are attributed to intrinsic as well as adaptive and acquired mechanisms (Lister et al., 2009; Moradali et al., 2017). The extensive and sometimes inappropriate use of antibiotics has supported the increase of multidrug resistance and therapeutic options against the already difficult-to-treat pathogen are becoming limited (Ventola, 2015). Therefore, alternative strategies to improve treatment are urgently needed.

*Pseudomonas aeruginosa* is a survival specialist since it adapts rapidly to challenging conditions such as iron depletion, phosphate starvation, heat, oxidative stress, competition from other microbiota, and host immune defenses (Hector et al., 2014; Winstanley et al., 2016). The expression of toxins enables *P. aeruginosa* to weaken the immune system, to harm host cells, and to cause substantial damage to tissue, thereby promoting dissemination. Toxins generate reactive oxygen species, degrade host proteins, hydrolyse lipids, and manipulate or block vital intracellular processes (Hauser, 2011; Gellatly and Hancock, 2013; Moradali et al., 2017). Extracellular toxins such as pyocyanin, alkaline proteases A, elastases LasA and LasB, and phospholipases C are largely released through type I or type II secretion systems, while other potent toxins such as ExoS, ExoT, ExoU, and ExoY are directly injected into the host cells through the type III secretion injectisome (Moradali et al., 2017). Additionally, *P. aeruginosa* expresses multiple other virulence determinants including surface-associated factors like flagella, type IV pili, alginate, or lipopolysaccharide. The plenitude and versatility of *P. aeruginosa* virulence determinants and variation in specificity across strains make it difficult to develop effective strategies to block or attenuate their detrimental effect. Possible strategies to combat the virulence of *P. aeruginosa* involve the manipulation of conserved regulatory networks connected to, e.g., quorum sensing (Smith and Iglewski, 2003; Jakobsen et al., 2013) or the stringent stress response (Mansour et al., 2016; Pletzer et al., 2017, 2018) to inhibit the expression of virulence factors.

In the study reported here, we used a different approach and aimed to sequester secreted toxins, thus, protecting host cells and tissues from damage. Recently, we demonstrated that non-toxic and non-immunogenic liposomes composed of sphingomyelin (Sm) and a high content of cholesterol (Ch:Sm; 66 mol/% Ch and 34 mol/% Sm) neutralized multiple toxins secreted by the Gram-positive bacteria *Streptococcus pneumoniae* and *Staphylococcus aureus*, thereby protecting host cells from deleterious plasma membrane damage (Henry et al., 2015; Wolfmeier et al., 2018). The liposomes were engineered to mimic lipid rafts, which are small (40 nm) microdomains in the plasma membrane of host cells enriched in sphingomyelin and cholesterol, thus representing preferred toxin target sites (Rosenberger et al., 2000). Since the liposomes sequestered structurally and functionally distinct bacterial toxins in Gram-positive bacteria, we hypothesized that they could exhibit broad-spectrum toxin binding capability. Therefore, we further investigated the potential of liposomes to sequester cytotoxins produced by the Gram-negative pathogen *P. aeruginosa*, to further attenuate its virulence.

We demonstrate here that the hypervirulent cystic fibrosis isolate LESB58 (Liverpool Epidemic Strain) secreted cytolytic factors under starvation conditions. Liposomal therapy attenuated the cytolytic activity of these secretomes toward human erythrocytes and the human bronchial epithelial cell line 16HBE14o-. Analyzing the cargo of liposomes incubated with the secretome, identified the hemolytic phospholipase C enzyme. Liposome-treated host cells and cutaneous abscesses showed decreased hemolytic activity and murine skin lesions, similar to an LESB58 *plcH* knockout mutant, indicating that PlcH can be targeted by engineered liposomes to reduce virulence of *P. aeruginosa*.

## Materials and Methods

### Liposomes

Unilamellar liposomes containing cholesterol:sphingomyelin (Ch:Sm, with 66 mol/% cholesterol, 40 mg/ml, diameter 130 nm) and sphingomyelin (Sm, 40 mg/ml, diameter 60 nm), both in sodium Tyrode’s buffer (140 mM NaCl, 5 mM KCl, 1 mM MgCl_2_, 2.5 mM CaCl_2_, 10 mM glucose, and 10 mM HEPES, pH = 7.4) were provided by Combioxin SA (Epalinges, Switzerland, product name CAL02).

### Bacterial Strains, Growth Conditions, and Supernatants

*Pseudomonas aeruginosa* LESB58 (Cheng et al., 1996), PAO1 (Stover et al., 2000), and PA14 (He et al., 2004) were used in this study. Bacteria were cultured in double yeast tryptone (dYT), Tryptic Soy Broth (TSB, Becton Dickinson), or modified synthetic cystic fibrosis medium (MSCFM, acid washed glassware; Palmer et al., 2007; Yeung et al., 2012) at 37°C under shaking conditions (250 rpm). To enhance the expression of *P. aeruginosa* virulence factors, iron and phosphate were reduced in MSCFM to 0.9 μM and 1 mM, respectively (Bains et al., 2012; Gellatly and Hancock, 2013). Cultures harboring individual vectors were supplemented with 15 μg/ml gentamicin (Gm) for *Escherichia coli* or 500 μg/ml Gm for LESB58. Bacterial growth was monitored using a spectrophotometer at an optical density of 600 nm (OD_600_).

To obtain bacterial supernatants, bacterial overnight cultures grown in MSCFM or dYT were washed once to remove iron or phosphate residuals and then inoculated at an OD_600_ of 0.1 in MSCFM or MEM tissue culture medium supplemented with 1% FBS and grown for 22–24 h at 37°C. Subsequently, bacteria were pelleted (5,000 × *g*, 10 min) and the resulting supernatants were filter sterilized (pore size 0.2 μm, Nalgene). When indicated, bacterial supernatants were centrifuged at high speed (100,000 × *g*) at 4°C for 1 h. The resulting supernatants were incubated with liposomes or sodium Tyrode’s buffer (vehicle) for 5 min with subsequent pelleting of the liposomes (100,000 × *g*) at 4°C for 1 h. The resulting liposome-free supernatants were used for the cytotoxicity assay, whereas the liposome/protein or vehicle/protein pellets were further analyzed *via* SDS-PAGE or mass spectrometry.

### PCR Amplifications and DNA Modifications

PCR primers are listed in Supplementary Table S1 PCR was carried out using the Phusion DNA polymerase (Thermo Scientific) in accordance with the manufacturer’s instructions and optimized annealing temperatures for each primer set. For PCR reactions performed with LESB58, bacterial cells were boiled at 98°C and subsequently pelleted at 13,000 rpm for 2 min. PCR reactions were supplemented with 5% dimethyl sulfoxide.

Restriction digestions were performed using Thermo Scientific FastDigest restriction enzymes according to the manufacturer’s instructions. All ligation reactions were carried out at room temperature using Thermo Scientific T4 DNA ligase. DNA purifications were either performed using the GeneJET PCR purification kit (Thermo Scientific) or the GeneJET Gel extraction kit (Thermo Scientific) following the manufacturer’s instructions.

### Construction of an Unmarked Phospholipase C Deletion Mutants in LESB58 and PAO1

The construction of the knockout vectors was carried out as previously described (Pletzer et al., 2014). Briefly, primers flanking the *plcH* gene with homologous overhang sequences were used to amplify the knockout alleles (approximately 500 bp). The obtained flanking fragments were used in an overlapping PCR reaction, the fusion fragment sub-cloned using the Zero Blunt TOPO kit (Invitrogen Life Technologies) and verified by sequencing before further transfer into the suicide vector pEX18Gm (Hoang et al., 1998) *via Bam*HI/*Hin*dIII. The deletion method was based on the site-specific insertional mutagenesis strategy of Schweizer and Hoang (1995) and carried out as described previously (Pletzer et al., 2014). Briefly, the suicide vector was transferred into *E. coli* ST18 and subsequently conjugated into LESB58 or PAO1. Single crossover mutants were selected on dYT agar plates containing 500 μg Gm (LESB58) or 50 μg Gm (PAO1) and resolved into unmarked deletions with 10% sucrose. Gene deletion was confirmed by locus-specific primers that bind outside the knockout alleles and sequencing of the obtained knockout fragment. The sequences between LESB58 and PAO1 showed 100% overlap. Strains used in this study are outlined in Supplementary Table S2.

### Complementation of the LESB58 *plcH* Mutant

The *plcH* gene including its upstream promoter region was PCR amplified from *P. aeruginosa* LESB58 genomic DNA. The PCR fragment was gel purified and cloned into *Hin*dIII/*Bam*HI restriction sites of plasmid pBBR1MCS-5 (Kovach et al., 1995), yielding pBBR5.*plcH* with additional ability to be expressed from the *lac* promoter. All constructs were sequenced before transformation into *P*. *aeruginosa* LESB58 wild type or Δ*plcH* as described previously (Pletzer et al., 2016).

### Human Cells

Blood from consenting healthy human volunteers was collected in sodium heparin anticoagulant collection tubes (BD Biosciences; following the University of British Columbia and University of Otago Human Ethics guidelines). Red blood cells (RBCs) were isolated as described previously (Wolfmeier et al., 2018). In brief, phosphate buffered saline (PBS, Thermo Fisher/Gibco) diluted blood was layered into Lymphoprep density gradient medium (STEMCELL Technologies), centrifuged (500 × *g* for 20 min), and the RBCs present in the bottom layer of the density gradient were washed three times with PBS and finally resuspended in Alsever’s solution (Sigma-Aldrich) for storage at 4°C (maximum 4 weeks).

The human bronchial epithelial cell line 16HBE14o- (HBE, RRID:CVCL_0112) was kindly provided by Dr. D. Gruenert (University of California San Francisco). HBE cells were maintained in MEM medium (Thermo Fisher/Gibco) supplemented with 10% FBS, 2 mML-glutamine (Thermo Fisher/Gibco), and 1% penicillin/streptomycin (Thermo Fisher/Gibco) at 37°C in 5% CO_2_ as described previously (Wolfmeier et al., 2018).

### Hemolysis Experiments

#### Liposome Hemolysis Assays

Red blood cells were washed three times with MSCFM (1,000 × *g*, 10 min). Liposomes were combined with bacterial supernatants (75–200 μl, as indicated) or MSCFM (vehicle, negative control) and 1% RBCs (total volume: 200 μl). RBCs were lysed with Triton X-100 (2% *v*/*v*, Sigma-Aldrich) serving as the positive control. After 1 h at 37°C, RBCs were centrifuged (1,000 × *g*, 10 min) and the hemoglobin content in the supernatant was assessed at OD_450_. Relative hemolysis (%) was calculated by $\frac{\Delta OD\;\mathrm{sample}-\Delta OD\;\mathrm{negative control}}{\Delta OD\;\mathrm{positive control}-\Delta OD\;\mathrm{negative control}}\times 100$ .

#### Phospholipase Hemolysis Assays

Red blood cells were treated with 100 μl of filter-sterilized supernatant from 24-h cultures of bacterial strains (wild type, phospholipase mutant, and complementation) grown in MEM tissue culture medium supplemented with 1% FBS at 37°C. Hemoglobin released from lysed erythrocytes was measured after pelleting intact cells and debris after 1 h incubation at 37°C *via* centrifugation (1,000 × *g*, 10 min). The supernatant was assessed at OD_450_ for each strain and normalized to control untreated RBCs and fully lysed RBCs (treated with 2% *v*/*v* Triton X-100).

For both experiments, a reference wavelength of 630 nm was used to eliminate non-specific absorbance (e.g., caused by the material).

### Cytotoxicity Assays

Cytotoxicity assays with HBE were performed as described previously (Wolfmeier et al., 2018). HBE cells (40,000) were seeded in MEM + 10% FBS 2 days before the assay (90% confluency at the day of the experiment). Just before treatment, the medium was replaced with MEM + 1% FBS (100 μl). Bacterial supernatants and liposomes or sodium Tyrode’s buffer (vehicle) were added as indicated (total reaction volume: 200 μl HBE cells) for 1 h at 37°C. The following controls applied as: Triton X-100 (2% *v*/*v*, Sigma-Aldrich) as the positive control, MSCF as the vehicle (negative control). After incubation, cells were centrifuged (500 × *g*, 5 min) and the lactate dehydrogenase (LDH) release was assessed with the Cytotoxicity Detection Kit^Plus^ (Roche) according to the manufacturer’s instructions. Relative LDH release (%) was calculated by $\frac{\Delta OD\;\mathrm{sample}-\Delta OD\;\mathrm{negative control}}{\Delta OD\;\mathrm{positive control}-\Delta OD\;\mathrm{negative control}}\times 100$ .

### Sodium Dodecyl Sulfate Polyacrylamide Gel Electrophoresis (SDS-PAGE) and Silver Staining

SDS-PAGE and silver staining were performed as described previously (Wolfmeier et al., 2018). For SDS-PAGE, samples were resuspended in 2 × SDS-loading buffer [65.8 mM Tris-HCl pH = 6.8, 26.3% (*w*/*v*) glycerol, 2.1% SDS, 0.01% bromophenol blue, and 355 mM 2-mercaptoethanol]. After heating the samples to 95°C (5 min), specimens were added to SDS-polyacrylamide gels (12% Mini-PROTEAN TGX stain-free precast gels, Bio-Rad; SDS-running buffer: 25 mM Tris, 192 mM glycine, and 0.1% SDS). SDS-PAGE was performed at 150 V for ~30 min. A protein standard (Precision Plus Protein Dual Color Standard, Bio-Rad) was included. For silver staining, the gel was incubated in 50% methanol until the next day. After a washing step (deionized water) and agitation in staining reagent (1.4 ml ammonium hydroxide, 21.0 ml of 0.36% NaOH, and 4.0 ml of 20% *w*/*v* AgNO_3_, topped up to 100 ml with deionized water) for 10 min, the gel was again washed and incubated in developer solution (2.5 ml citric acid (1% *w*/*v*), 0.25 ml formaldehyde (38% *v*/*v*), topped up to 250 ml with deionized water). After reaching a good staining intensity, the gel was incubated with 50% methanol/10% acetic acid and subsequently imaged (ChemiDoc Touch Imaging System, Bio-Rad).

### Mass Spectrometry

Mass spectrometric analysis of liposome/toxin pellets was performed as described previously (Wolfmeier et al., 2018). Gel lanes (35 μl/sample) were excised and cut into small fragments. In gel, digestion was performed as previously described (Shevchenko et al., 1996) and was performed by the Proteomics Core Facility of the University of British Columbia (Vancouver, BC, Canada). In brief, the gel pieces were washed with 50% digestion buffer (50 mM NH_4_HCO_3_) and 50% EtOH dehydrated with absolute EtOH. After the incubation with dithiothreitol (10 mM) at 56°C for 45 min and with iodoacetamide (55 mM) for 30 min at room temperature, the samples were washed with digestion buffer. Samples were dehydrated and vacuum centrifugation was applied to remove the remaining EtOH. After incubating the specimens in trypsin solution (12.5 ng/μl) at 37°C overnight, acetic acid (neat) was added, samples were rigorously mixed. Gel extraction was repeated three times with 0.5% AcOH, 30% MeCN, 0.5% AcOH, and 100% MeCN. Vacuum centrifugation was applied to remove organic constituents. Desalting was performed on C18 STAGE tips (Rappsilber et al., 2007) eluted with 80% acetonitrile, dried and suspended in 3% acetonitrile +0.1% formic acid. An Agilent 6550 QToF mass spectrometer equipped with an Agilent 1200 capillary HPLC, connected by a 2.1 mm × 250 mm POROShell C18 column, was loaded with up to 5 μg of protein. QToF was set to AutoMS/MS mode, at 2 spectra/s for MS, and 3 spectra/s for MS/MS scans. MaxQuant 1.5.3.30 was used to analyze the LC–MS/MS data (default values for Agilent QToF data including 1% FDR) against the Uniprot *P. aeruginosa* LESB58 (UP000001527) database.

### Phospholipase C Enzymatic Assay

To perform the phospholipase C activity assay in a 96-well plate, based on Luberto et al. (2003), we added 40 μl of *p*-nitrophenylphosphorylcholine (*p*-NPPC, 37.5 mM, in 100 mM Tris–HCl at pH 7.4, 25% glycerol) to 60 μl of bacterial supernatant samples pre-incubated without or with liposomes (after removal of liposomes *via* centrifugation at 45,000 × *g* for 10 min prior to the assay). The absorbance of the chromogenic product *p*-nitrophenyl at 405 nm (Stonehouse et al., 2002; 60 s interval, 37°C) was measured progressively starting immediately after adding *p*-NPPC.

### Murine Cutaneous Infection Model

All mice used in this study were female outbred CD-1, 7 weeks of age and weighed ~25 ± 3 g at the time of the experiment. Animals were purchased from Charles River Laboratories (Wilmington, MA). We used up to 3% isoflurane to anesthetize the mice and euthanized them with carbon dioxide. The full characterization of this murine model has been previously described (Pletzer et al., 2017).

Briefly, *P. aeruginosa* LESB58 strains were grown to an OD_600_ of 1.0 in dYT broth, cells washed and adjusted to an OD_600_ of 1.0 again in sterile PBS. The injection volume was 50 μl, which was ~1–5 × 10^7^ CFU. The bacterial suspension was injected into the right side of the dorsum. All utilized liposomes were tested for skin toxicity prior to efficacy testing. Treatment was applied directly into the subcutaneous space into the infected area (100 μl) at 1 h post-infection. The progression of the disease/infection was monitored daily, and abscesses (visible swollen and inflamed lumps) were measured on day three using a caliper. Skin abscesses were excised (including all accumulated pus), homogenized in sterile PBS using a Mini-Beadbeater-96 (Biospec products) for 5 min and bacterial counts determined by serial dilution. Experiments were performed at least three times independently with two to four animals per group.

### Ethics Statement

Animal experiments were performed in accordance with the Canadian Council on Animal Care (CCAC) guidelines and were approved by the University of British Columbia Animal Care Committee (protocol A14-0363). The number of mice was based on power calculations and our previous experience using this animal model.

### Quantification and Statistical Analysis

Plots were generated and statistical significance analyzed using Graph Pad Prism v9.00. All details (statistical test, number of experiments, and definition of significance) are provided in the corresponding figure legends.

## Results

### Liposomes Diminished the Cytolytic Activity of *Pseudomonas aeruginosa* Supernatants

**Figure 1:**
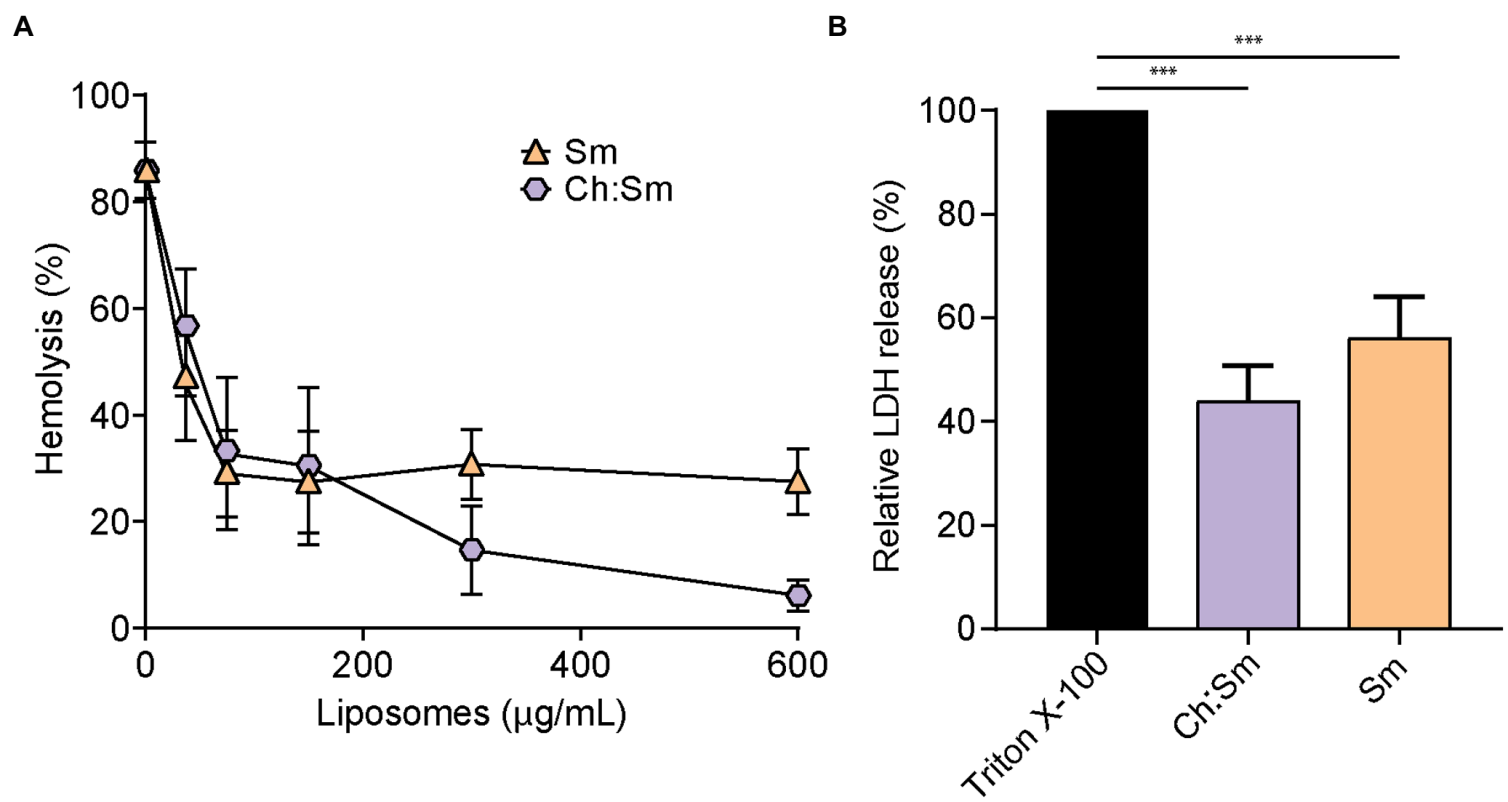
Engineered liposomes reduced the lysis of human cells induced by *Pseudomonas aeruginosa* bacterial supernatant grown in modified cystic fibrosis medium. **(A)** Human red blood cells were incubated with LESB58 (100 μl) bacterial supernatant and cholesterol-containing (Ch:Sm) or sphingomyelin only (Sm) liposomes for 1 h (*n* = 11, each treatment). **(B)** 16HBE14o- cells after challenge with *Pseudomonas aeruginosa* LESB58 stationary-grown supernatants (100 μl), normalized to Triton X-100. Both types of liposomes (300 μg/ml) decreased the release of lactate dehydrogenase (*n* = 17). Error bars, mean ± standard error. A one-way ANOVA with *post-hoc* Dunn’s multiple comparison test was performed between treated and non-treated exposure, value of *p* (^***^ < 0.001) adjusted for multiple comparisons.

To investigate whether engineered liposomes exhibited protective effects against secreted *P. aeruginosa* virulence factors, LESB58 was cultured in modified synthetic cystic fibrosis medium until late stationary phase. Incubation of filter-sterilized supernatants (100 and 150 μl) with 1% RBCs indicated that treatment with Sm liposomes reduced hemolysis in a concentration-dependent manner, by up to 27% at the highest concentration tested (600 μg; Figure 1A). While a similar trend was observed for Ch:Sm liposomes when 100 μl of supernatants was used, reaching 6% at the highest concentration (600 μg; Figure 1A), the protection was diminished when LESB58 supernatants were increased to 150 μl (Supplementary Figure S1). Each liposome type was further used to investigate cell protection by assessing release of lactate dehydrogenase from bacterial supernatant challenged HBE cells (100 μl). Both Ch:Sm and Sm liposomes (300 μg) significantly reduced LDH release by 44 and 56%, respectively (Figure 1B). When supernatants from the traditional laboratory *P. aeruginosa* PAO1 and PA14 strains were used, a comparable, but variable concentration-dependent reduction in hemolysis and significant reduction in LDH release from HBE cells was also observed with Ch:Sm and Sm liposomes (Supplementary Figure S2).

### The *Pseudomonas aeruginosa* Virulence Factor Phospholipase C Was Bound by Liposomes

**Figure 2:**
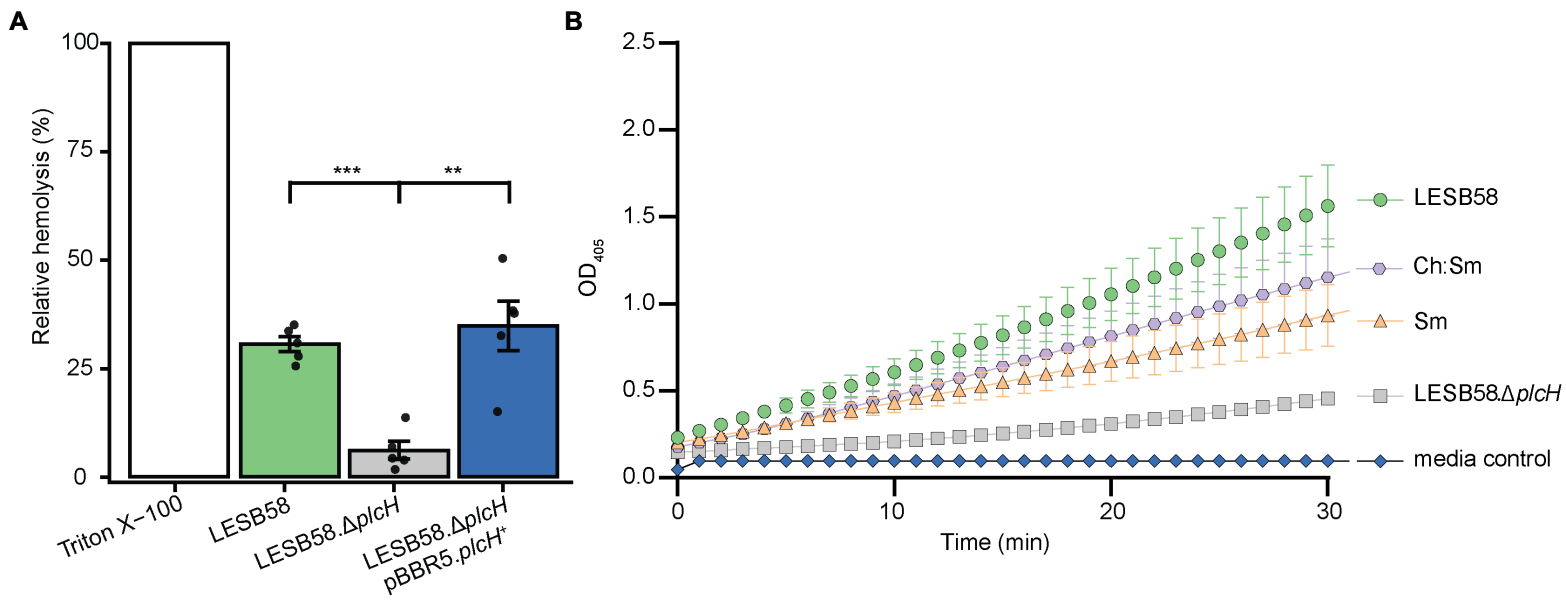
*Pseudomonas aeruginosa* supernatant-induced hemolysis is attenuated by the removal of phospholipase C and supernatants pre-incubated with liposomes have reduced hemolytic phospholipase C activity. **(A)** RBCs were treated with 100 μl of filter-sterilized supernatant from 24-h cultures of bacterial strains grown in MEM + 1% FBS incubated at 37°C. OD_450_ was measured for each strain and normalized to control untreated RBCs and fully lysed RBCs (treated with 2% *v*/*v* Triton X-100). One-way ANOVA with *post-hoc* Dunnett test, ^**^Bonferroni correct value of *p* < 0.01, and ^***^Bonferroni corrected value of *p* < 0.001, *n* = 5, error bars represent ±SE. **(B)** The hemolytic phospholipase C driven hydrolysis of colorless *p-*nitrophenylphosphorylcholine to yellow *p-*nitrophenol was assessed for supernatants pre-treated with 300 μg/ml cholesterol-containing (Ch:Sm) and sphingomyelin only (Sm) liposomes or vehicle control (LESB58 wild type, LESB58.Δ*plcH*; six independently prepared wild-type supernatants for LESB58, Sm, Ch:Sm; three independently prepared LESB58.Δ*plcH* bacterial supernatants). Error bars are mean ± SE; SE not shown for mutant and media control due to very small variation. A two-way ANCOVA with *post-hoc* Bonferroni value of *p* adjustment was carried out using the wild-type (LESB58) phospholipase C activity as a control. All conditions were significantly different, adjusted value of *p* of 0.031 for Ch:Sm, and *p* < 0.0001 for all other conditions.

To further identify which proteins/toxins are potentially sequestered by the liposomes, we investigated whether cytolysins, which are responsible for cellular damage, were bound to the liposomes. Therefore, bacterial supernatants were pre-incubated with 600 μg Ch:Sm or Sm liposomes and ultra-centrifuged to pellet the liposomes to analyze their cargo. The liposome/protein or liposomes/vehicle control pellets were applied to SDS gel electrophoresis, which revealed several liposome-bound proteins (Supplementary Figure S3). To further identify the liposome-bound bacterial proteins, we applied mass spectrometry to liposome pellets. Since Sm liposomes showed more consistent hemolytic protection, we only focused on this liposome type here. Mass spectrometry revealed 12 *P. aeruginosa* proteins including a chaperonin (GroEL), phosphodiesterase (GlpQ), Phospholipase C (PlcH), reductoisomerase (IlvC), outer membrane proteins (OprM and OprO), carbamoyltransferase (ArcB), lipoprotein (OprI), aminopeptidase, phosphatase, and two hypothetical proteins (Table 1). Of these proteins, phospholipase C (PlcH) was identified as the only characterized virulence factor bound to the liposomes and was thus selected for further investigation. To identify if this was an appropriate target, we first created a clean deletion mutant in *plcH* (PALES_44741; LESB58.Δ*plcH*) and then performed hemolysis experiments. The mutant showed significantly reduced hemolytic activity (23.9% total reduction) against RBCs compared to the wild type and complementation (Figure 2A). Reduced hemolysis was also confirmed in a *plcH* clean deletion mutant in PAO1 (PA0844; 39.2% reduction) and in a transposon mutant of PA14, *plcH*::MAR2xT7 (29% reduction; Supplementary Figure S4).

**Table 1:** Complete list of *Pseudomonas aeruginosa* proteins found in the bacterial supernatant/liposome pellet.

Protein	Gene Name	Locus Tag	Size (kDa)	Locus Tag (PAO1 ortholog)
Chaperonin GroEL	*groEL*	PALES_47641	57	PA4385
Glycerophosphoryl diester phosphodiesterase	*glpQ*	PALES_03441	42	PA0347
Hemolytic phospholipase C	*plcH*	PALES_44741	83	PA0844
Hypothetical protein	n.a.	PALES_48751	25	PA4495
Hypothetical protein	n.a.	PALES_12461	42	PA3734
Ketol-acid reductoisomerase	*ilvC*	PALES_50791	36	PA4694
Major intrinsic multiple antibiotic resistance efflux outer membrane protein OprM	*oprM*	PALES_04251	53	PA0427
Ornithine carbamoyltransferase	*arcB*	PALES_55641	38	PA5172
Outer membrane lipoprotein OprI	*oprI*	PALES_22111	9	PA2853
Putative aminopeptidase	n.a.	PALES_21241	58	PA2939
Putative phosphatase	n.a.	PALES_24701	74	PA2635
Pyrophosphate-specific outer membrane porin OprO	*oprO*	PALES_17861	48	PA3290

n.a., not available.

A *p*-nitrophenylphosphorylcholine (NPPC) assay was performed to confirm that liposomes could bind and potentially neutralize PlcH secreted by *P. aeruginosa*. Bacterial supernatants of LESB58 and the corresponding phospholipase C mutant (Δ*plcH*) were incubated with the liposomes Ch:Sm or Sm or vehicle control. Liposomes were subsequently removed by centrifugation. Liposomal pre-treatment led to a significant reduction of PlcH activity in the supernatants indicating that PlcH had been bound by both types of liposomes (Figure 2B). PlcH in *P. aeruginosa* PAO1 and PA14 supernatants was also bound and neutralized by both types of liposomes (Supplementary Figure S5).

### Liposomes Attenuated *Pseudomonas aeruginosa* Virulence in a Murine Subcutaneous Abscess Model

**Figure 3:**
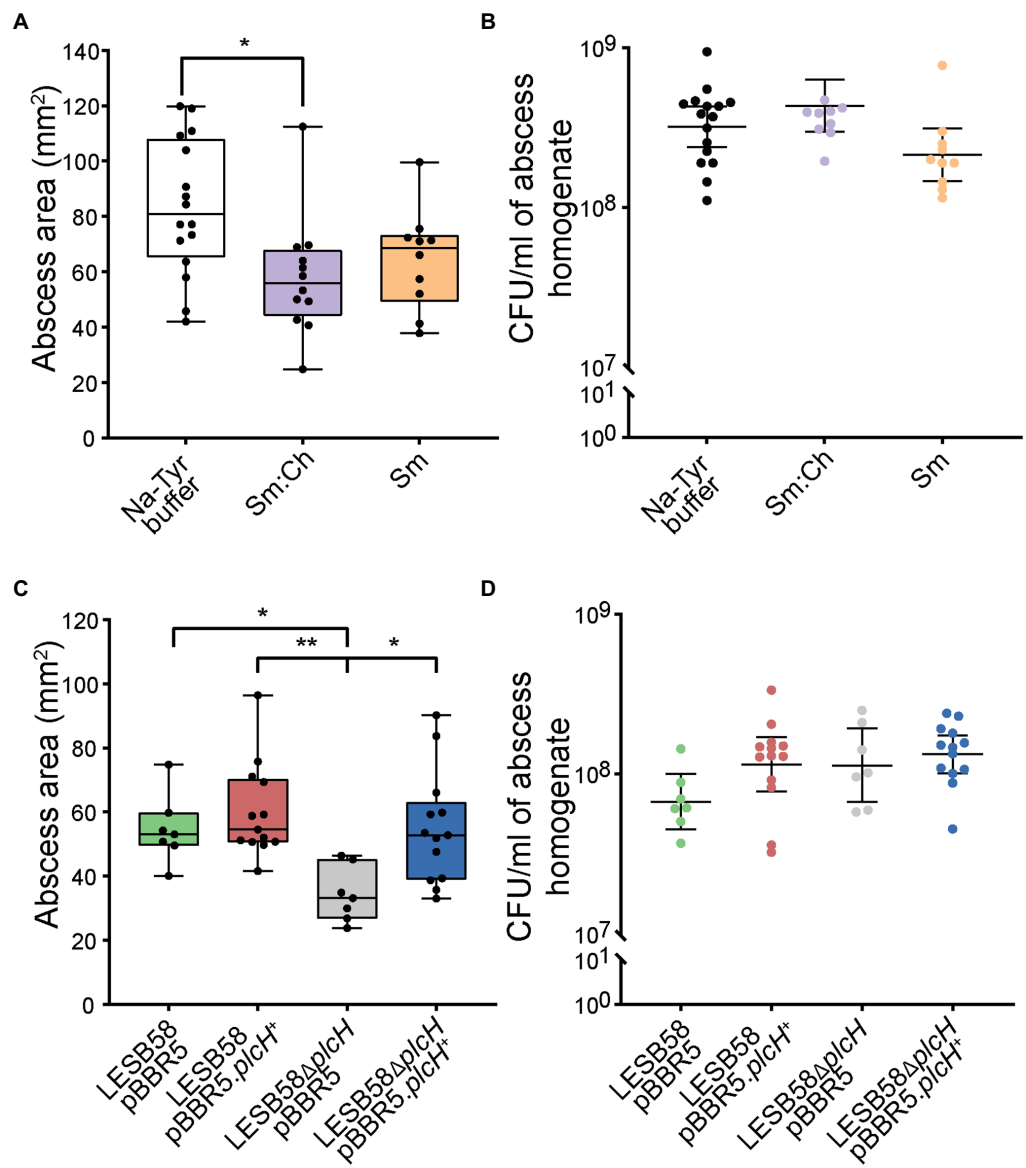
Effects of liposome treatment and phospholipase knockout mutant in a *Pseudomonas aeruginosa* LESB skin abscess mouse model. CD-1 mice were subcutaneously infected with a high bacterial density (5 × 10^7^ CFU) of LESB58 wild type **(A,B)**, LESB58 carrying an empty vector pBBR5 or overexpression vector pBBR5.*plcH*^+^, and phospholipid mutant LESB58.Δ*plcH* carrying an empty vector pBBR5 or overexpression vector pBBR5.*plcH*^+^ **(C,D)**. Lesion sizes (Box and whiskers plot) and CFU counts (with geometric mean) were determined 3 day post-infection. Liposome treatment (top) and mutant studies (bottom)—**(A,C)** abscess size measurements, and **(B,D)** bacterial counts per abscess. All experiments were done at least three times independently with 2–4 mice/group. Statistical analysis was performed using one-way ANOVA, Kruskal-Wallis test with Dunn’s correction. The asterisk indicates a significant difference to the wild type (^*^p < 0.05; ^**^p < 0.01).

Phospholipases have been implicated in tissue damage (Fiester et al., 2016) and their inhibition has been suggested to impair virulence (Flores-Diaz et al., 2016). Since liposomes may bind and neutralize the virulence factor PlcH, we further tested their potential *in vivo*. We have previously shown that liposomes can be safely delivered subcutaneously (Wolfmeier et al., 2018). Using a murine cutaneous high-density (>10^7^ CFU) *P. aeruginosa* abscess model, we show that both Sm and Ch:Sm liposomes decreased abscess size by 17 and 32%, respectively. The effect of Ch:Sm liposomes was statistically significant, and while the result with Sm liposomes was not significant, a clear trend to smaller abscesses was observed (Figure 3A). As anticipated, neither of the liposomes reduced the bacterial burden (Figure 3B), similar to the result previously observed for *S. aureus* (Wolfmeier et al., 2018).

We further investigated the importance of *P. aeruginosa* phospholipase C in the abscess model and found that the *plcH* mutant demonstrated significantly reduced abscess size (37%), while maintaining similar bacterial loads at the infection site (Figures 3C,D), equivalent to the impact of Ch:Sm liposomes. Complementation of the mutant restored abscess size to wild-type levels, while overexpression had no significant impact on disease development. This indicates that PlcH may be targeted by liposomes to reduce virulence of *P. aeruginosa* in a mouse model.

## Discussion

The production of extracellular virulence factors from bacteria is critical in establishing and promoting dangerous infections. Virulence factors act as bacterial attack weapons and help to overcome innate and adaptive host immune responses. The release of these important components can not only induce tissue damage but also trigger a life-threatening cytokine storm (Le Berre et al., 2011; Strateva and Mitov, 2011; D’Elia et al., 2013; Newman et al., 2017; Azam and Khan, 2019). Blocking or attenuating virulence factors in bacterial infections shows promise as a combination treatment with traditional antibiotics (Fleitas Martinez et al., 2019; Buroni and Chiarelli, 2020; Vlaeminck et al., 2020; Wang et al., 2020; Ogawara, 2021) and may also increase the hosts immune system’s ability to clear the infection (Cegelski et al., 2008; Rasko and Sperandio, 2010). Here, we demonstrated that liposomes neutralized the cytolytic virulence factor PlcH secreted by the multidrug-resistant pathogen *P. aeruginosa* LESB58. Liposomal treatment significantly reduced red blood cell lysis, epithelial cell damage, and skin tissue abscesses, which together suggests that liposome treatment can be used to tackle Gram-negative *P. aeruginosa* infections.

Treatment with liposomes led to attenuation of cytotoxicity against human bronchial epithelial cells and reduced hemolysis against human red blood cells. Supernatants from *P. aeruginosa* were less effective at causing cell lysis in the presence of liposomes in a dose-dependent manner (Figure 1; Supplementary Figures S1, S2). It is well established that liposome treatment can reduce cytotoxicity and hemolysis through sequestering exotoxins produced by many Gram-positive bacteria (Henry et al., 2015; Wolfmeier et al., 2018; Wang et al., 2019; Ayllon et al., 2021), including *S. aureus*, *Streptococcus pyogenes*, *S. pneumoniae*, and *Clostridium perfringens* (Henry et al., 2015). We have previously shown that sphingomyelin (Sm) and the combination of sphingomyelin (Sm) and 66 mol/% cholesterol (Ch:Sm) effectively sequestered cholesterol-dependent cytolysins, phenol-soluble modulins (PSM-α3), α-hemolysin (Wolfmeier et al., 2018), and phospholipase C (Henry et al., 2015). We further hypothesized that virulence factors (toxins) produced by Gram-negative bacteria could also be targeted *via* sphingomyelin-containing liposomes. Indeed, we found a significant reduction of *P. aeruginosa*-induced cell damage when treated with either Ch:Sm and Sm liposomes, although the latter showed a larger effect (Figure 2B; Supplementary Figures S1, S2). Additionally, *P. aeruginosa* supernatants were less hemolytic when treated with liposomes (Figure 1; Supplementary Figure S2). To identify whether certain cytolysins were sequestered by (bound to) liposomes leading to reduced hemolysis, mass spectrometry of *P. aeruginosa* supernatants incubated with liposomes was performed. This approach revealed that the exotoxin hemolytic phospholipase C (encoded by the *plcH* gene) was a cytolysin bound by Sm liposomes (Table 1). The release of phospholipases during infection contributes to the degradation of phospholipid-rich mucus layers, cleavage of phospholipids from pulmonary surfactants, and host cell membrane damage (Schmiel and Miller, 1999; Ochsner et al., 2002; Barker et al., 2004; Flores-Diaz et al., 2016). Administration of hemolytic phospholipase C in mice has been shown to cause extensive and significant cell damage (Meyers et al., 1992). Treatment of human cells in culture has shown rapid cytolytic activity when given PlcH and reduced PlcH production leads to impaired virulence of *P. aeruginosa* and reduced ability to maintain infections (Ostroff et al., 1989; Meyers et al., 1992; Wargo et al., 2011). This strongly suggests that the removal or sequestration of PlcH during infection might have beneficial effects for patients. It is well established that PlcH is important in maintaining virulence during chronic *P. aeruginosa* infections, and production of antibodies against PlcH are commonly identified in persons with cystic fibrosis (Granstrom et al., 1984; Hollsing et al., 1987).

To further investigate whether we could target this important virulence factor released from a Gram-negative pathogen, we incubated *P. aeruginosa* cell supernatants with Sm and Ch:Sm liposomes; both significantly reduced hemolytic phospholipase C activity in a quantitative assay (Figure 2; Supplementary Figure S4). This strongly supports our hypothesis that toxins (in this case PlcH from *P. aeruginosa*) may be targeted with engineered liposomes. There have been extensive studies into the use of liposomes as conjugates for antibiotic delivery to reduce toxicity, increase tissue and biofilm penetration, and to reduce antibiotic resistance development (Haworth et al., 2019; Wang et al., 2019; Bassetti et al., 2020; Bilton et al., 2020; Ibaraki et al., 2020). This research suggests that liposome itself can enhance the effect of treatment of *P. aeruginosa* infections, attenuating the virulence factor PlcH.

Our results obtained with PlcH from *P. aeruginosa* LESB58 was also observed for strains PAO1 and PA14 but showed strain-dependent variability in effects of total hemolysis when comparing wild type and mutant (Figure 2A; Supplementary Figure S4). We assume this relates, at least partially, to the different supernatant compositions of these strains. Consistent with this, the LESB58 supernatant was much better neutralized at lower liposome concentrations than was the PAO1 or PA14 supernatants. The different compositions and efficiencies of liposomes in binding different cytotoxins could explain the difference in hemolysis, despite no differences in the relative efficiency versus HBE cells. Cholesterol influences the binding and activity of cholesterol-dependent cytolysins and other toxins. It is not present in bacteria, and thus represents a target of choice that is more host-like. On the other hand, cholesterol-containing bilayers are more rigid than those without cholesterol and would decrease the binding affinity of toxins (such as phospholipase) that penetrate the bilayer. Therefore, cholesterol-containing liposomes are more effective with cholesterol-binding toxins, whereas sphingomyelin liposomes are tailored to the phospholipid bilayer-binding toxins. This is important, as cytolytic components from *P. aeruginosa* affect different types of mammalian cells differently, and also suggests the effect of PlcH sequestration by liposomes may not be unique to the hyper virulent clinical isolate LESB58 but may be broadly applicable to different strains of *P. aeruginosa* and possibly other Gram-negative bacteria. In chronic *P. aeruginosa* infections, the reduction in exotoxins and virulence factors is theorized to contribute to immune system evasion, thus, enabling the infection to persist (Bragonzi et al., 2009; Cullen and McClean, 2015; Winstanley et al., 2016; Wardell et al., 2021). In addition, secreted phospholipase C has been shown to suppress the neutrophil respiratory burst, which may also facilitate *P. aeruginosa* survival (Terada et al., 1999). In contrast to many exotoxins and virulence factors, the prevalence of PlcH across *P. aeruginosa* has been shown to be retained in >90% of clinical isolates (Lanotte et al., 2004; Ellappan et al., 2018; Pournajaf et al., 2018; Bogiel et al., 2020), suggesting that PlcH is not commonly lost during infection, or that it is required for continuing infection (PlcH is potentially produced in small amounts in chronic infections). This seeming conservation of PlcH makes it an excellent therapeutic target to reduce the virulence of *P. aeruginosa* during infection. A previous study by Wargo et al. (2011) showed that mice infected with an isogenic *P. aeruginosa plcHR* mutant had less respiratory distress and better lung function when compared to WT-infected mice, while there were no differences in the bacterial load. This correlates well with our chronic skin infection model where we show the importance of PlcH for full virulence in causing tissue damage and abscess formation. Wargo et al. (2011) further used miltefosine, a phospholipid drug, to reduce PlcH-dependent surfactant function, providing evidence that targeting PlcH *in vivo* is a promising approach to treat *P. aeruginosa* and to protect lung function. Successfully targeting *P. aeruginosa* PlcH might also have other advantages such as, for example, reducing vascular lesions, improving wound healing, and decreasing sepsis (Vasil et al., 2009).

Overall, our *in vivo* data for Sm:Ch liposomes correlated well with our *in vitro* findings (32% reduction in abscess size and 44% reduction in HBE viability), although there was a bigger gap for Sm liposomes. The results could have many explanations including accessibility to toxins *in vivo* (based on the diffusibility of the liposomes in the skin infection model), the more complex mixture of mammalian cells and extracellular components in the animal model, as well as the relative production of small molecule toxins such as pyocyanin, phenazines, pyoverdine, or metalloproteases such as AprA (Pletzer et al., 2020). In conclusion, our study reveals the interaction between liposomes and hemolytic phospholipase C in *P. aeruginosa*. Reducing the virulence of *P. aeruginosa* infections with liposome treatment can help the natural immune response to adapt and better fight off infection. We acknowledge that there might be other important factors bound by the liposomes that have yet to be studied. Neutralizing virulence factors to disarm bacteria is a promising strategy to change the course of disease as evidenced in this study leading to smaller abscesses and reduced tissue damage. While liposome treatment may sequester PlcH, it remains to be studied if multiple liposome applications or coupling liposomes with conventional antibiotic treatment regimens could lead to synergistic effects and better treat *P. aeruginosa* or other Gram-negative infections. Since phospholipases are present in many other important Gram-negative bacteria such as *Burkholderia* or *Bordetella* (Wargo et al., 2011; Srinon et al., 2020), as well as *Mycobacterium*, liposome treatment may offer a broad-spectrum strategy to tackle the virulence of these pathogens during infection.

## Data Availability Statement

The original contributions presented in the study are included in the article/**Supplementary Material**, further inquiries can be directed to the corresponding authors.

## Ethics Statement

Animal experiments were performed in accordance with the Canadian Council on Animal Care (CCAC) guidelines and were approved by the University of British Columbia Animal Care Committee (protocol A14-0363).

## Author Contributions

HW, DP, AD, EB, and RH contributed to conception and design of the study. HW, SW, LL, RF, and DP performed the experiments and statistical analysis. HW, SW, and DP wrote the first draft of the manuscript and wrote sections of the manuscript. All authors contributed to manuscript revision, read, and approved the submitted version.

## Funding

Funding from the Canadian Institutes for Health Research FDN-154287 to RH is gratefully acknowledged. The content is solely the responsibility of the authors and does not necessarily represent the official views of the Canadian Institutes for Health Research. RH holds a Canada Research Chair and a UBC Killam Professorship. HW received an Early Postdoc Mobility fellowship from the Swiss National Science Foundation under Award Number P2BEP3_165401 and DP received a Cystic Fibrosis Canada postdoctoral fellowship.

## Conflict of Interest

The authors declare that the research was conducted in the absence of any commercial or financial relationships that could be construed as a potential conflict of interest.

## Publisher’s Note

All claims expressed in this article are solely those of the authors and do not necessarily represent those of their affiliated organizations, or those of the publisher, the editors and the reviewers. Any product that may be evaluated in this article, or claim that may be made by its manufacturer, is not guaranteed or endorsed by the publisher.

## Supplementary Material

The Supplementary Material for this article can be found online at: https://www.frontiersin.org/articles/10.3389/fmicb.2022.867449/full#supplementary-material
