# Supplementary material for: Targeting the Pseudomonas aeruginosa Virulence Factor Phospholipase C With Engineered Liposomes
Source: Front Microbiol. 2022 Mar 18;13:867449. doi: 10.3389/fmicb.2022.867449 (PMC8971843; doi:10.3389/fmicb.2022.867449)
Supplement: Supplementary file 1 [file Data_Sheet_1.pdf]

Supplementary Figures

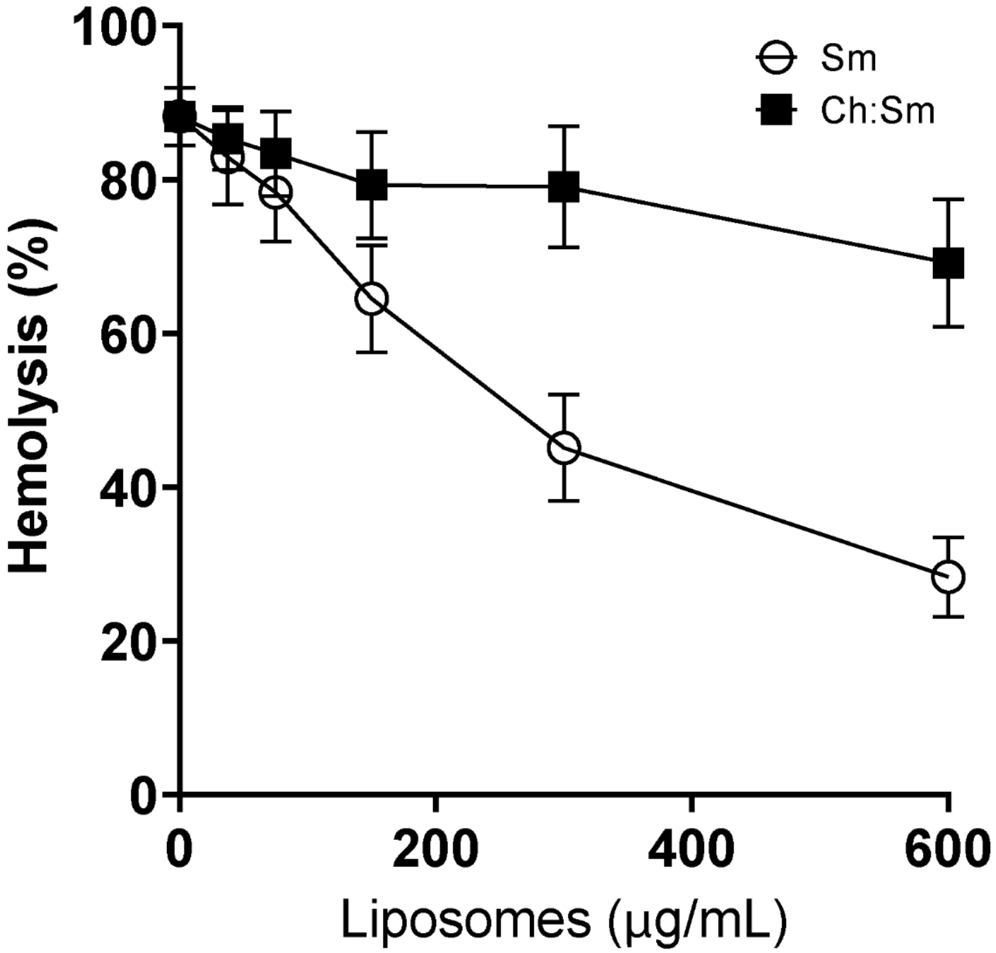

Fig S1: Engineered liposomes reduced the lysis of human cells induced by *P. aeruginosa* bacterial supernatant grown in modified cystic fibrosis medium. Human red blood cells were incubated with stationary-phase grown LESB58 (150 µl) supernatants and cholesterol-containing (Ch:Sm) or sphingomyelin only (Sm) liposomes for 1h (n=11, each treatment).

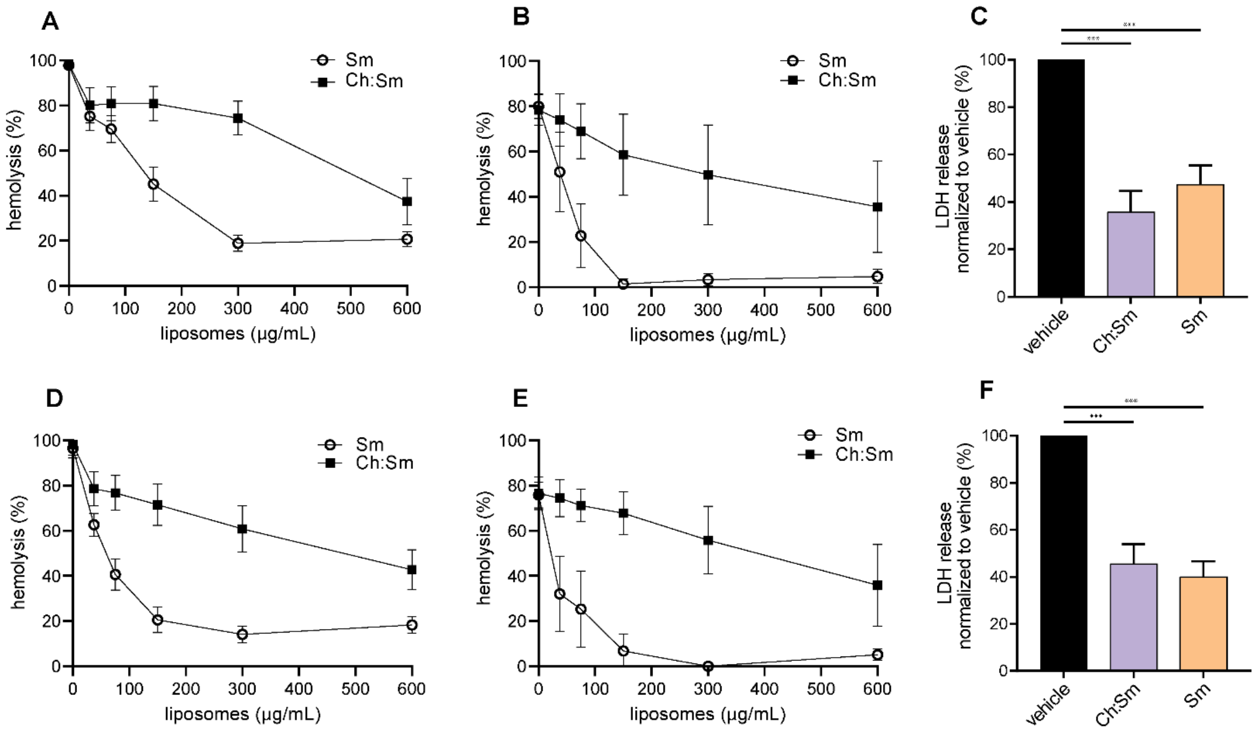

Fig S2: Engineered liposomes reduced the lysis of human cells induced by stationary-phase grown *P. aeruginosa* supernatants in modified cystic fibrosis medium. (A) Human red blood cells were incubated with PA14 (75 µl) supernatant and liposomes (Ch:Sm or Sm) for 1h (n=7, each treatment). (**B**) Human red blood cells were incubated with PA14 (200 µl) bacterial supernatant and liposomes cholesterol-containing (Ch:Sm) or sphingomyelin only (Sm) liposomes for 1 h (n=4, each treatment). (**C**) 16HBE14o- cells after 1 h challenge with *P. aeruginosa* PA14 supernatant. Both types of liposomes (300 µg/ml) decreased the release of lactate dehydrogenase (n=18). Error bars, mean + SEM. A one-way ANOVA with post-hoc Dunn's multiple comparison test was performed between treated and non-treated exposure, p-value reported adjusted for multiple comparisons. (**D**) Human red blood cells were incubated with PAO1 (75 µl) bacterial supernatant and liposomes (Ch:Sm or Sm) for 1h (n=8, each treatment). (**E**) Human red blood cells were incubated with PAO1 (200 µl) bacterial supernatant and liposomes cholesterol-containing (Ch:Sm) or sphingomyelin only (Sm) liposomes for 1h (n=6, each treatment). (**F**) 16HBE14o- cells after challenge with *P. aeruginosa* PAO1 supernatant. Both types of liposomes (300 µg/ml) decreased the release of lactate dehydrogenase (n=18). Error bars, mean + SEM. A one-way ANOVA with post-hoc Dunn's multiple comparison test was performed between treated and non-treated exposure, *p*-value (*** < 0.001) reported adjusted for multiple comparisons.

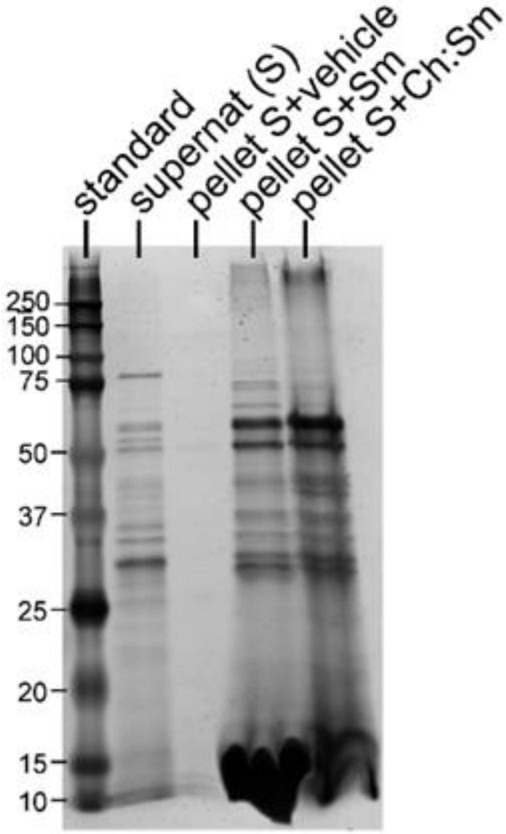

Fig S3: *P. aeruginosa* proteins bound by liposomes. Silver-stained SDS-PAGE of LESB58 supernatants and pellets of supernatants (S) pre-incubated with buffer (vehicle), cholesterol-containing liposomes (Ch:Sm) or sphingomyelin (Sm) liposomes. Representative image of three bacterial supernatant preparations per strain and condition.

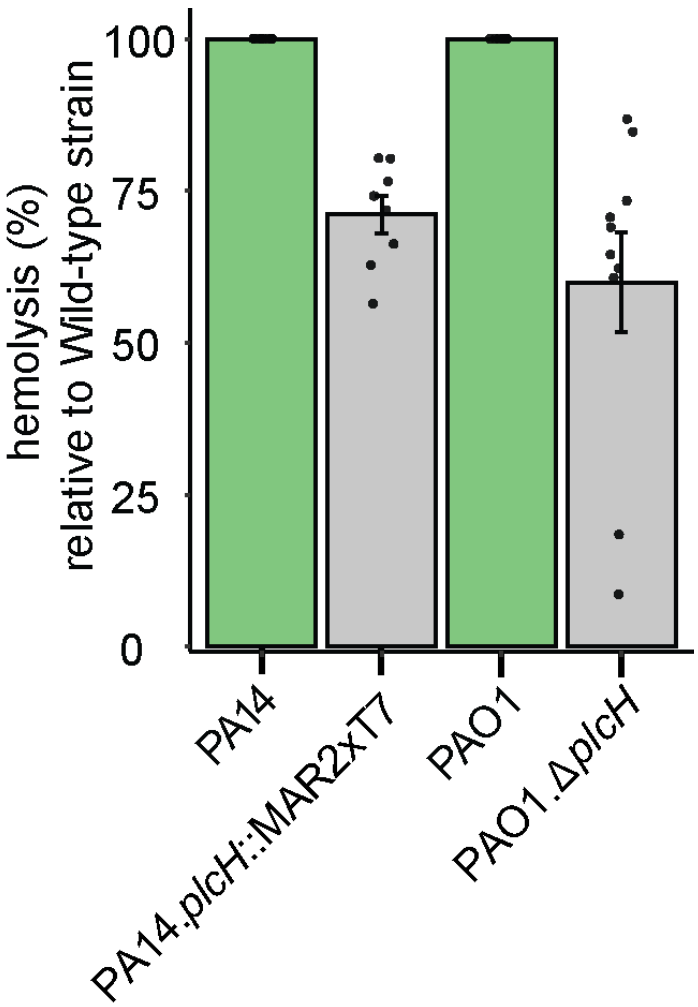

Fig S4: ***P. aeruginosa*** **PA14 and PAO1** ***plcH*** **mutants showed reduced hemolysis.** Human red blood cells were treated with 100 µL of filter-sterilized supernatant from 24-hour cultures of bacterial strains grown in MSCFM incubated at 37°C. OD_{450} was measured for each strain and normalised to untreated red bloods cells and wild-type hemolysis as complete (100%) hemolysis.

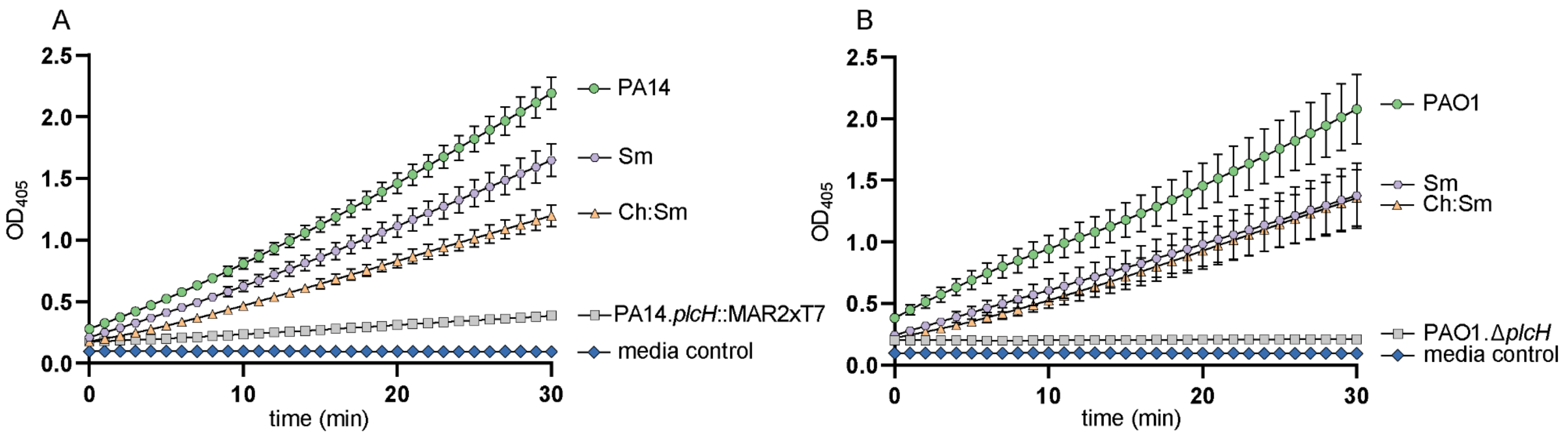

Fig S5: *P. aeruginosa* supernatants pre-incubated with liposomes demonstrated reduced hemolytic phospholipase C activity. The hemolytic phospholipase C driven hydrolysis of colourless *p*-nitrophenylphosphorylcholine to yellow *p*-nitrophenol was assessed for supernatants pre-treated with 600 µg/mL cholesterol-containing (Ch:Sm) or sphingomyelin only (Sm) liposomes or vehicle control (wt, Δ*plcH*) (six independently prepared wild-type supernatants for wild-type, Sm, Ch:Sm; three independently prepared *plcH* mutant supernatants). Error bars, mean ± SEM. (**A**) shows *P. aeruginosa* PA14 and (**B**) shows *P. aeruginosa* PAO1. A two-way ANOVA with post-hoc Tukey's multiple comparison test was carried out using the wild-type phospholipase C activity as a control (PA14 or PAO1). All conditions were significantly different, adjusted *p*-value <0.0001. (**C**) RBCs were treated with 100 µL of filter-sterilized supernatant from 24-hour cultures of bacterial strains grown in MSCFM incubated at 37°C. OD_{450} was measured for each strain and normalised to control untreated RBCs and wild-type hemolysis as complete hemolysis.

Supplementary Tables

Table S1: Primers used for generation of *plcH* knockout in *P. aeruginosa* LESB58. Small letters indicate the incorporated restriction sites and italics the homologous overlapping region.

**Primer**	**Sequence (5' – 3')**
plcH-up-fwd(BamHI)	agaggatccACCTCGCGCTTGAGCTGTT
plcH-up-rev	CCACCCGGGAAATAAAACG*AGCGAGGAGTCCATCGCAT*
plcH-down-fwd	*ATGCGATGGACTCCTCGCT*CGTTTTATTTCCCGGGTGG
plcH-down-rev(HindIII)	gccaagctTGCCTCGAAAGCGACTATC
plcH-out-fwd	TCCAGATCTCGAAGTCGAGC
plcH-out-rev	GATGAACTTCCAGACCGGAG
plcH-oe_fwd(HindIII)	atcgaagcttAAGATCAGCGAAATCGGCGG
plcH-oe_rev(BamHI)	atcaggatccTCAGGTCGCTGCGATGTCGA

Table S2: Strains used in this study.

**Reference**	***P. aeruginosa* Strain**	**Details**
1	LESB58	Liverpool Epidemic Strain, from CF patient
2	PAO1	Standard *P. aeruginosa* reference strain, isolated 1954, from wound
3	PA14	Standard *P. aeruginosa* virulent reference strain, isolated from burn wound
This study	LESB58 pBBR5	LESB58 containing pBBR5 cloning vector
This study	LESB58 pBBR5.*plcH*$^{+}$	LESB58 containing a pBBR5 vector with functional PlcH expression
This study	LESB58Δ*plcH* pBBR5	LESB58 lacking functional PlcH through site-specific mutagenesis. Containing pBBR5 cloning vector
This study	LESB58Δ*plcH* pBBR5.*plcH*$^{+}$	LESB58 lacking functional PlcH through site-specific mutagenesis. Containing a pBBR5 vector with functional PlcH expression
This study	PAO1Δ*plcH*	PAO1 lacking functional PlcH through site-specific mutagenesis.
4	PA14.*plcH*::MAR2xT7	PA14 *plcH* transposon mutant

1. Cheng K, Smyth RL, Govan JR, Doherty C, Winstanley C, Denning N, et al. Spread of beta-lactam-resistant *Pseudomonas aeruginosa* in a cystic fibrosis clinic. Lancet. 1996;348(9028):639-642. doi: 10.1016/S0140-6736(96)05169-0.
2. Stover CK, Pham XQ, Erwin AL, Mizoguchi SD, Warrener P, Hickey MJ, et al. Complete genome sequence of *Pseudomonas aeruginosa* PAO1, an opportunistic pathogen. Nature. 2000;406(6799):959-964. doi: 10.1038/35023079.
3. He J, Baldini RL, Deziel E, Saucier M, Zhang Q, Liberati NT, et al. The broad host range pathogen *Pseudomonas aeruginosa* strain PA14 carries two pathogenicity islands harboring plant and animal virulence genes. Proc Natl Acad Sci U S A. 2004;101(8):2530-2535. doi: 10.1073/pnas.0304622101.
4. Liberati NT, Urbach JM, Miyata S, Lee DG, Drenkard E, Wu G, Villanueva J, Wei T, and Ausubel FM. 2006. An ordered, nonredundant library of *Pseudomonas aeruginosa* strain PA14 transposon insertion mutants. Proc Natl Acad Sci U S A103:2833–2838.
